# Cold-induced degradation of core clock proteins implements temperature compensation in the *Arabidopsis* circadian clock

**DOI:** 10.1126/sciadv.adq0187

**Published:** 2024-09-27

**Authors:** Akari E. Maeda, Hiromi Matsuo, Tomoaki Muranaka, Norihito Nakamichi

**Affiliations:** Graduate School of Bioagricultural Sciences, Nagoya University, Furo-cho, Chikusa, Nagoya 464-8601, Japan.

## Abstract

The period of circadian clocks is maintained at close to 24 hours over a broad range of physiological temperatures due to temperature compensation of period length. Here, we show that the quantitative control of the core clock proteins TIMING OF CAB EXPRESSION 1 [TOC1; also known as PSEUDO-RESPONSE REGULATOR 1 (PRR1)] and PRR5 is crucial for temperature compensation in *Arabidopsis thaliana*. The *prr5 toc1* double mutant has a shortened period at higher temperatures, resulting in weak temperature compensation. Low ambient temperature reduces amounts of PRR5 and TOC1. In low-temperature conditions, PRR5 and TOC1 interact with LOV KELCH PROTEIN 2 (LKP2), a component of the E3 ubiquitin ligase Skp, Cullin, F-box (SCF) complex. The *lkp2* mutations attenuate low temperature–induced decrease of PRR5 and TOC1, and the mutants display longer period only at lower temperatures. Our findings reveal that the circadian clock maintains its period length despite ambient temperature fluctuations through temperature- and *LKP2*-dependent control of PRR5 and TOC1 abundance.

## Teaser

Maintenance of *Arabidopsis* circadian clock against temperature fluctuations relies on the quantity control of clock proteins.

## INTRODUCTION

The circadian clock is an internal timekeeping system that governs the diel rhythms of diverse physiological processes in cyanobacteria, fungi, plants, and animals (*1*, *2*). While individual core clock genes vary among these kingdoms, there are conserved characteristics. One of them is temperature compensation, by which circadian clocks maintain their periodicity at around 24 hours against fluctuating ambient temperatures. Temperature compensation is a counterintuitive phenomenon because chemical reaction rates are generally temperature dependent (*3*). About seven decades ago, Hasting and Sweeny (*4*) have hypothesized that temperature-dependent activation of period-lengthening factors underpins temperature compensation.

Several molecular components are known to contribute to temperature compensation. The reconstituted in vitro cyanobacterial KaiC phosphorylation rhythm maintains a period of about 24 hours despite ambient temperature fluctuations, but its exact molecular mechanism is unknown (*5*, *6*). Mutations in Neurospora casein kinase 2 (CK2) and a mutation in the FREQUENCY (FRQ) phosphorylation site by CK2 show similar temperature compensation phenotypes, suggesting that FRQ phosphorylation by CK2 is involved in temperature compensation (*7*). The *Drosophila* PERIOD (PER) phosphorylation and nuclear localization protein have been shown to affect temperature compensation (*8*, *9*). The mammalian CK1 phosphorylation of two PER2 phosphosites differentially modulates PER2 degradation (*10*), and binding affinity between CK1 and its substrates balanced by temperature is also implicated in temperature compensation (*11*). On the basis of current knowledge, posttranslational regulation of proteins appears to be a key element in temperature compensation across these organisms.

In *Arabidopsis*, an intriguing phenotype was found in mutants lacking both *PSEUDO-RESPONSE REGULATOR 9* (*PRR9*) and *PRR7*. This double mutant showed an overcompensation phenotype, in which the circadian period was lengthened at high growth temperatures (*12*). The DNA binding affinity of a Myb-type transcription repressor CIRCADIAN CLOCK-ASSOCIATED 1 (CCA1) is attenuated by CK2 phosphorylation and is involved in temperature compensation (*13*). *REVEILLE* (*RVE*) genes encoding Myb-type transcription activators also affect temperature compensation (*14*). The period lengths of the *rve8 rve6 rve4* triple mutant change more notably at lower temperatures compared to the wild type, highlighting the importance of *RVE* genes in period tuning under these conditions. Compensatory mutations in *ZEITLUPE* (*ZTL*) and *GIGANTEA* (*GI*) may modulate temperature compensation (*15*). Specifically, a pair of *ZTL* and *GI* alleles in Cvi accession enables the maintenance of the clock pace at higher temperatures (27°C). In addition to specific steps, the network balance controlled by CRYPTOCROME (CRY) signaling is involved in temperature compensation by regulating amounts of core clock proteins such as LHY (*16*).

In this study, we found that temperature-dependent quantity control of PRR5 and TIMING OF CAB EXPRESSION 1 (TOC1, also known as PRR1) is crucial for temperature compensation. Mutants lacking *PRR5* and *TOC1* (*prr5 toc1*) displayed significantly shortened periods at higher temperatures, indicating weak temperature compensation. Our phenotypic analyses suggested that the activity of *PRR5* and *TOC1* is reduced at lower temperatures, which is consistent with the fact that PRR5 and TOC1 proteins are ubiquitinated and degraded at lower temperatures. Affinity proteomics analysis revealed that both PRR5 and TOC1 associate with LOV KELCH PROTEIN 2 (LKP2), a component of the E3 ubiquitin ligase Skp, Cullin, F-box (SCF) complex, particularly at lower temperatures. *LKP2* was found to be required for low temperature–induced PRR5 and TOC1 protein reduction and critical for period maintenance specifically at low temperatures. Overall, the temperature-dependent quantity control of PRR5 and TOC1 by LKP2 is indispensable for temperature compensation.

## RESULTS

### PRR5 and TOC1 are required to maintain circadian period length at high temperature

**Fig. 1.**
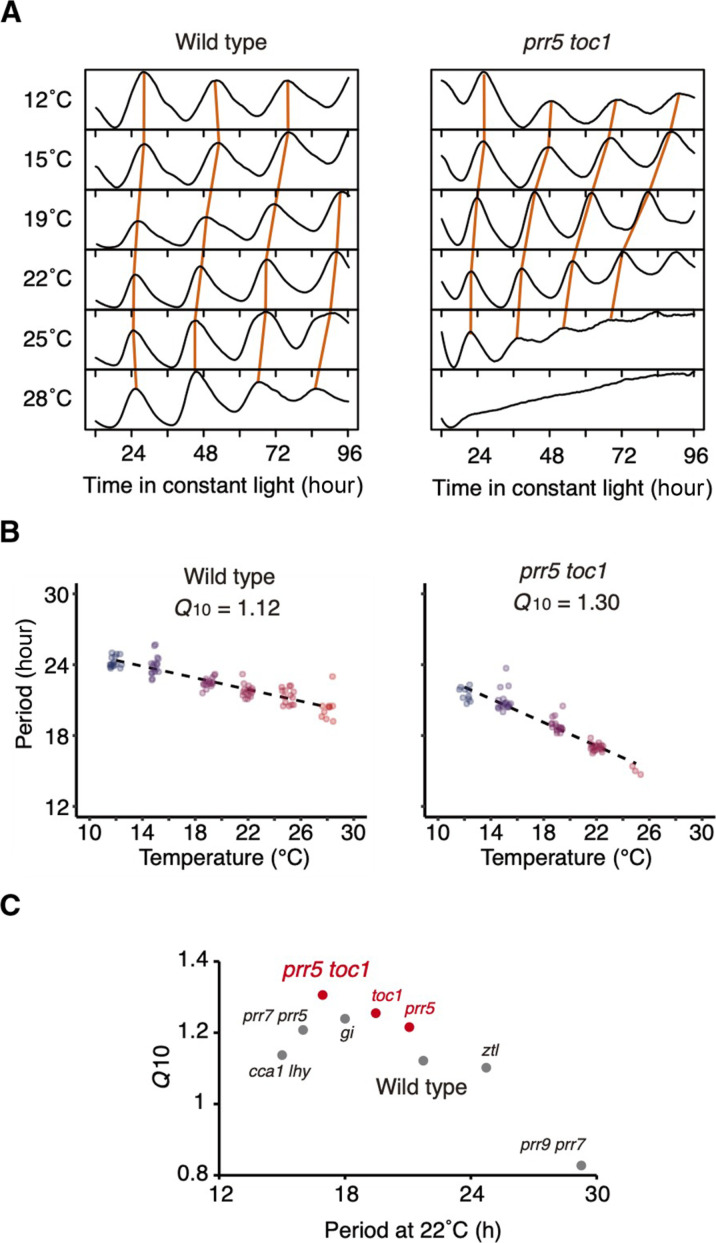
Circadian rhythms in period mutants grown at various temperatures. (**A**) Mean traces of 16 seedlings expressing the *CCA1:LUC* reporter in the wild type and in *prr5 toc1*. (**B**) Period length and *Q*_10_ values of wild type and *prr5 toc1*. Note that a sample with a fitting error value exceeding 0.05 was discarded due to incorrect period determination (*n* = 10 to 16 for wild type and *n* = 3 to 20 for *prr5 toc1*). (**C**) *Q*_10_ values of period mutants. Traces and period lengths of the mutants are shown in fig. S1.

To understand the mechanisms underlying temperature compensation in the circadian clock, we measured circadian rhythms of *CCA1:LUC* in the period mutants—*cca1 lhy*, *prr9 prr7*, *prr7 prr5*, *prr5 toc1*, *gi*, and *ztl*—at temperatures ranging from 12° to 28°C under constant white light conditions (Fig. 1A and fig. S1A). Period length in wild type is shortened by about 2.9 hours from 12° to 25°C (24.21 ± 0.12 hours at 12°C and 21.34 ± 0.18 hours at 25°C) (*12*). The calculated *Q*_10_ value (i.e., the change in the rate of a reaction resulting from an increase in temperature of 10°C) (*12*) was 1.12 in wild type, while *Q*_10_ in the period mutants ranged from a low of 0.82 in *prr9 prr7* as previously reported (*12*) to a high of 1.30 in *prr5 toc1* (Fig. 1, B and C, and fig. S1). In *prr5 toc1*, period length was shortened by approximately 4.5 hours at 25°C compared to 12°C (21.52 ± 0.18 hours at 12°C and 17.00 ± 0.07 hours at 25°C (Fig. 1, A and B), suggesting that *PRR5* and *TOC1* are involved in temperature compensation. *PRR5* and *TOC1* encode transcriptional repressors of the PRR family that share target genes in the circadian clock (*17*). *prr5* and *toc1* single mutants showed a short period, suggesting that these genes act as period-lengthening factors at 22°C (*17*). The *Q*_10_ of *prr5* and *toc1* single mutants were 1.21 and 1.23, respectively, intermediate between wild type and *prr5 toc1* double mutants, suggesting an additive function of *PRR5* and *TOC1* in temperature compensation (Fig. 1C and fig. S1). PRR5 and TOC1 proteins are targeted by ZTL, a component of the E3 ubiquitin ligase SCF complex, for degradation especially in the dark (*17*). In addition, accession-specific genetic interactions between *ZTL* and *GI* affect temperature compensation under constant red light conditions (*15*). In our study, the *Q*_10_ of *ztl-3* mutants was 1.10 and similar to that of wild type (Fig. 1C and fig. S1). However, the period length of *ztl-3* at 12°C was shorter than that at 15°C, suggesting an overcompensated phenotype around the lower temperature range (fig. S1). The *Q*_10_ of *gi-2* was 1.25, suggesting involvement of *GI* for temperature compensation (Fig. 1C and fig. S1), as previously demonstrated (*18*).

### PRR5 and TOC1 activity decreases at lower temperatures

**Fig. 2.**
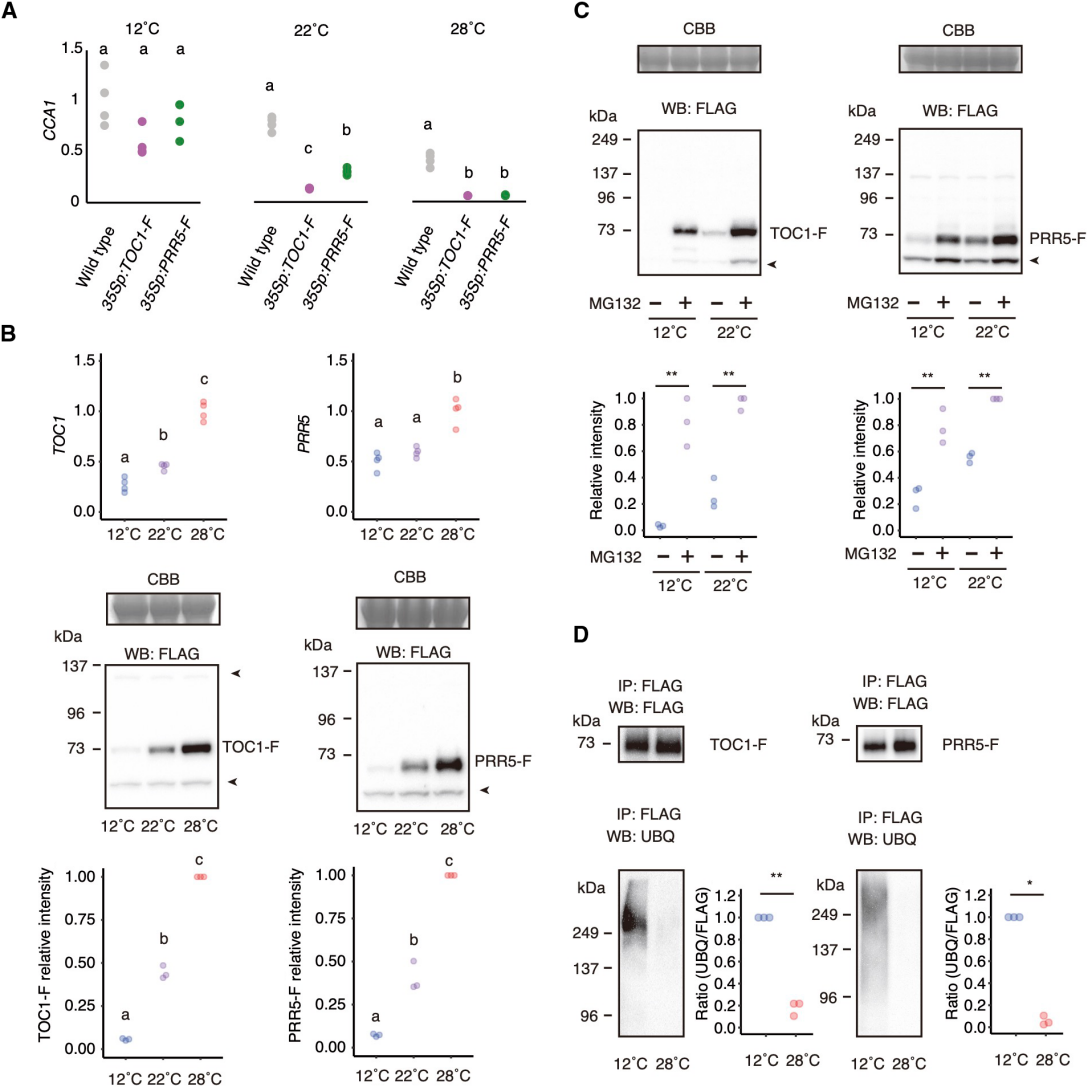
Quantitation of *CCA1* expression and PRR5 and TOC1 proteins in plants incubated at 12°, 22°, and 28°C. (**A**) Relative expression of *CCA1* at 12°, 22°, and 28°C (four biological replicates). The mean value of *CCA1* relative to *IPP2* in the wild type at 12°C was normalized to 1.0. (**B**) *TOC1* mRNA levels in *35Sp:TOC1-F* and *PRR5* in *35Sp:PRR5-F* seedlings 24 hours after temperature shifts to 12°, 22°, and 28°C (top). TOC1-F and PRR5-F protein accumulation in seedlings at 12°, 22°, and 28°C. Coomassie brilliant blue (CBB)–stained gels and Western blot probed with anti-FLAG antibody (WB: FLAG) are shown. TOC1-F or PRR5-F band intensity of three biological replicates (bottom graphs). (**C**) TOC1-F and PRR5-F protein accumulation in seedlings treated with 26*S* proteasome inhibitor MG132. (**D**) Ubiquitination of TOC1-F or PRR5-F proteins in seedlings at 12°, 22°, and 28°C. Protein samples immunoprecipitated with anti-FLAG antibody (IP: FLAG) were detected with anti-FLAG (WB: FLAG) or anti-ubiquitin (WB: UBQ) antibodies. The band intensity of the strongest signal in the gel was normalized to 1. Letters in the graphs show statistical differences as determined by Tukey-Kramer test. Double and single asterisks indicate statistical differences *P* < 0.01 and *P* < 0.05, respectively, as determined by Student’s *t* test. Arrow heads indicate non-specific bands.

We noticed that the *prr5 toc1* double mutant exhibited both low amplitude and short periods at 25°C, and rhythmicity was lost at 28°C (Fig. 1A). Thus, *PRR5* and *TOC1* appear to be more important for the circadian clock at higher temperatures. This led us to speculate that *PRR5* and *TOC1* functions are enhanced at high temperatures. To test this, we analyzed the expression of *CCA1*, a typical target of PRR5 and TOC1, in plants expressing a C-terminal triple FLAG (F)–tagged PRR5 or TOC1 under the control of the constitutive Cauliflower Mosaic Virus promoter (*35Sp:PRR5-F* and *35Sp:TOC1-F*, respectively) (Fig. 2A). The seedlings grown under 12-hour light and 12-hour darkness at a constant temperature of 22°C for 7 days after germination were transferred into constant light at various temperatures and sampled after 24 hours. Sampling time was subjective dawn. As previously reported (*19*, *20*), *CCA1* expression at 22°C was decreased to 40 and 20% in *35Sp:PRR5-F* and *35Sp:TOC1-F* seedlings, respectively, when compared to the wild type around subjective dawn (Fig. 2A). *CCA1* expression at 28°C was decreased to about 10% in both *35Sp:PRR5-F* and *35Sp:TOC1-F* compared to wild type (Fig. 2A). However, at 12°C, *CCA1* expression in both *35Sp:PRR5-F* and *35Sp:TOC1-F* plants was comparable to wild type. Thus, the effects of overexpression of *PRR5* and *TOC1* on *CCA1* expression are enhanced with increasing temperatures. The amount of *PRR5* and *TOC1* mRNA in the *35Sp:PRR5-F* and *35Sp:TOC1-F* seedlings around subjective dawn was comparable at 12° and 22°C (fig. S2). Therefore, the mRNA levels of *TOC1* and *PRR5* are unlikely to be the major reason for the temperature activation of their function.

Hypocotyl length is regulated by the circadian clock through the modulation of phytochrome signaling (*21*). Overexpression of either *PRR5* or *TOC1* results in a shorter hypocotyl compared to the wild type at 22°C, particularly under short day conditions (*22*). The hypocotyl lengths of *35Sp:PRR5-F* and *35Sp:TOC1-F* seedlings were 69 and 54%, respectively, compared to that of the wild type at 22°C under 8-hour light/16-hour dark conditions (fig. S3). At 28°C, the hypocotyl lengths of *35Sp:PRR5-F* and *35Sp:TOC1-F* were 36 and 26%, respectively, compared to that of the wild type (fig. S3). The hypocotyl lengths of all seedlings were similar at 12°C, likely due to attenuation of phyB signaling by cold temperature (fig. S3) (*23*). No conclusion regarding PRR5 and TOC1 activities at 12°C could be made from the results. However, hypocotyl lengths at 22° and 28°C support our hypothesis that *PRR5 and TOC1* functions are enhanced at higher temperatures.

### PRR5 and TOC1 are degraded at low temperature

To investigate the molecular mechanisms underlying the temperature-dependent effects on *PRR5* and *TOC1* activities, we analyzed the levels of exogenously expressed TOC1-FLAG (TOC1-F) and PRR-FLAG (PRR5-F) proteins in *35Sp:TOC1-F* and *35Sp:PRR5-F* plants. Seedlings grown under constant light at 22°C were transferred and kept for 24 hours at 12°, 22°, or 28°C. Increasing the temperature from 22° to 28°C for 24 hours caused a 2.9-fold increase of TOC1-F protein amounts (Fig. 2B) and a 2.2-fold increase of *TOC1* mRNA amounts (fig. S3). Decreasing the temperature from 22° to 12°C resulted in a 75% reduction of TOC1-F amounts and a 41% reduction of *TOC1* mRNA levels (Fig. 2B and fig. S3). Temperature shift generally entrains the clock; thus, it is possible that the circadian phase shift induced by the temperature change affects *TOC1* mRNA and protein levels. To test this, we analyzed TOC1-F protein and *TOC1* mRNA in samples collected 30 and 36 hours after the temperature change. A temperature change to 28°C for 30 and 36 hours caused 1.6- and 1.8-fold increase of *TOC1* mRNA and 3.7- and 3.8-fold increase of TOC1-F protein compared to the 22°C condition. A temperature shift to 12°C for 30 and 36 hours caused a 48 and 30% reduction of *TOC1* mRNA and a 65 and 28% reduction of TOC-F protein compared to 22°C. *TOC1* mRNA under the control of the 35*S* promoter increased about two to four times, and TOC1-F protein increased 5 to 17 times at 28°C compared to 12°C. The TOC1-F amount changes were less dependent on time after temperature shifts. Likewise, increasing the temperature from 22° to 28°C for 24 hours resulted in a 2.8-fold increase of PRR5-F protein amounts, whereas decreasing the temperature to 12°C resulted in a 90% reduction (Fig. 2B). Amounts of *PRR5* mRNA in seedlings at 28°C for 24 hours were about twice those at 12° or 22°C (fig. S3). Amounts of exogenously expressed *PRR5* mRNA and PRR5-F protein were increased about 2-fold and 8- to 14-fold at 28°C compared to 12°C, and these increases were less affected by time after the temperature shifts. Note that an increase of PRR5 protein at 28°C compared to 20°C was previously shown in *PRR5pro:PRR5-FLAG* plants (*24*). To further examine the temperature-dependent change of TOC1 protein amount, we analyzed the TOC1–yellow fluorescent protein (YFP) fusion protein in the *35Sp:TOC1-YFP* line. TOC1-YFP amounts increased with temperature, whereas green fluorescent protein (GFP) amounts in *35Sp:GFP* plants did not vary significantly at 12°, 22°, or 28°C (fig. S4). These results indicate that ambient temperature affects protein levels of PRR5 and TOC1.

Increased protein degradation may account for reduced PRR5 and TOC1 accumulation at low temperatures. To test this, seedlings grown under constant white light at 22°C were treated with the 26*S* proteasome inhibitor MG132 and incubated at 12°C for 24 hours. MG132 treatment resulted in a 25-fold increase of TOC1-F amounts at 12°C (Fig. 2C). As control experiments, MG132 treatment at 22°C resulted in a 3.6-fold increase of TOC1-F at 22°C. PRR5-F protein amounts were increased threefold at 12C° and 1.8-fold at 22°C upon treatment by MG132 (Fig. 2C), suggesting that the 26*S* proteasome protein degradation pathway is essential for temperature-dependent regulation of PRR5 and TOC1 levels at 12°C. We then investigated the ubiquitination of PRR5 and TOC1 by Western blotting using an anti-ubiquitin antibody (Fig. 2D). *35Sp:TOC1-F* and *35Sp:PRR5-F* seedlings grown at 22°C under constant light conditions were treated with MG132 to inhibit 26*S* proteasome-dependent protein degradation and further incubated at 12° or 28°C for 24 hours. TOC1-F and PRR5-F proteins extracted from the seedlings were immunoprecipitated with the anti-FLAG antibody, and ubiquitination of TOC1-F and PRR5-F was quantified using the anti-ubiquitin antibody. The intensity of the signal corresponding to ubiquitinated TOC1-F from seedlings incubated at 12°C was 5.5 times more intense than that at 28°C (Fig. 2D). Low temperature–enhanced ubiquitination was also observed with PRR5-F (17.4-fold increase at 12°C compared to 28°C; Fig. 2D). These results indicate that ubiquitination of PRR5 and TOC1 is enhanced by low temperature.

### LKP2 is required for reducing the levels of PRR5 and TOC1 at low temperature

**Fig. 3.**
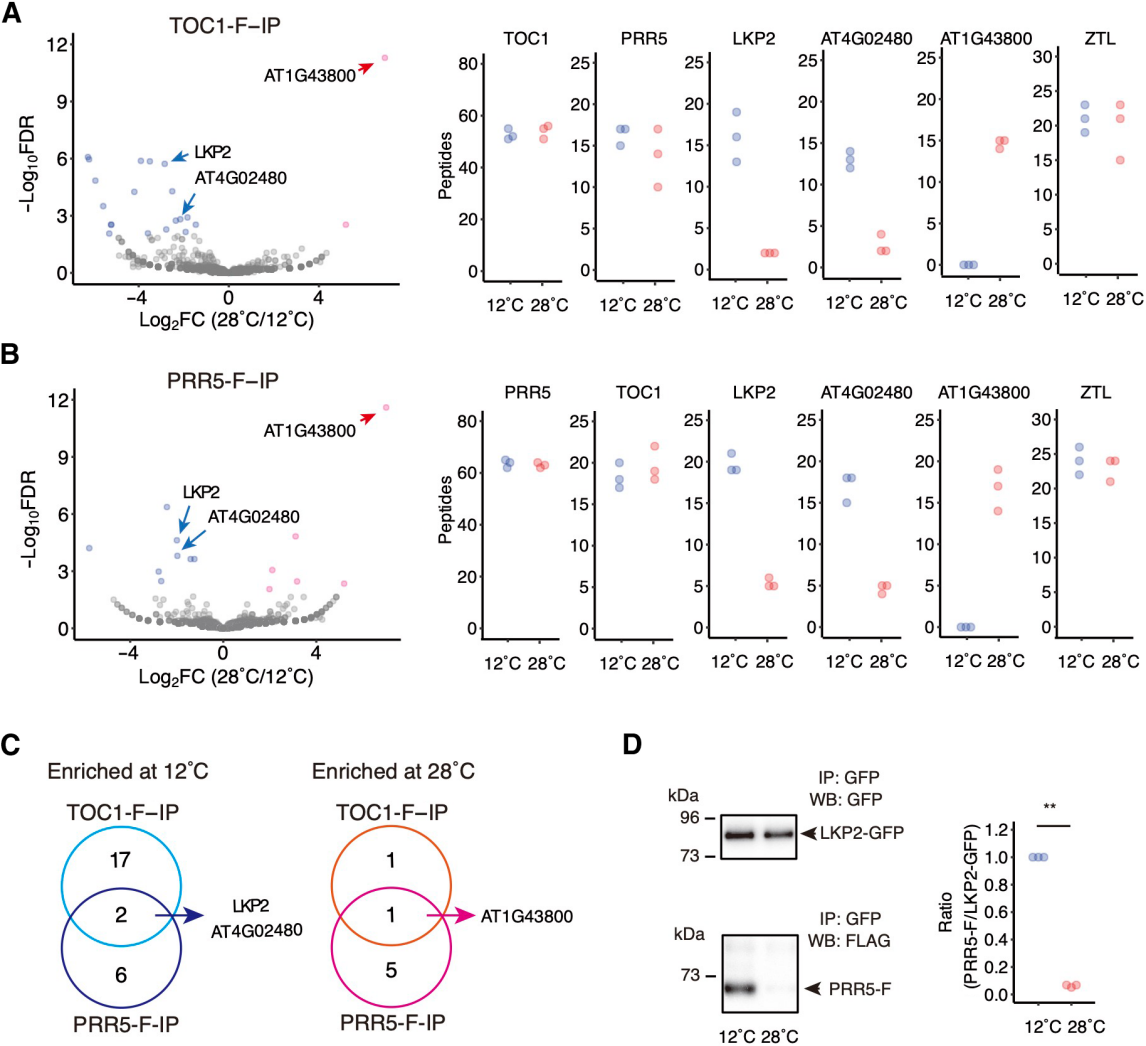
Interaction of PRR5 and TOC1 proteins with LKP2. (**A**) Volcano plot of proteins in the TOC1-F–immunoprecipitated (IP) fraction from plants grown at 12°C versus 28°C, analyzed by LC-MS/MS (left). Three independent biological replicates were used. The *x* axis shows the log 2 fold change (FC) in plants at 28°C versus 12°C. The *y* axis shows the log_10_ of the false discovery rate (FDR) value. Number of detected peptides corresponding to TOC1, PRR5, LKP2, AT4G02480, AT1G43800, and ZTL in TOC1-F–immunoprecipitated fractions from plants grown at 12° or 28°C (right). (**B**) Volcano plot of PRR5-F–immunoprecipitated fraction from plants grown at 28°C versus 12°C (left), and the six proteins in PRR5-F–immunoprecipitated fractions (right). (**C**) Venn diagrams showing number of proteins enriched in TOC1-F– and PRR5-F–immunoprecipitated fractions in plants grown at 12° or 28°C. (**D**) LKP2-GFP protein was immunoprecipitated from *35Sp:PRR5-F*/*lkp2-cr3*/*LKP2-GFP* plants grown at 12° or 28°C, and LKP2-GFP and PRR5-FLAG proteins in immunoprecipitated fractions were detected (left). Three independent biological replicates were performed for the quantification (right). The band intensity of the 12°C sample was normalized to 1.

To elucidate the molecular mechanisms underlying PRR5 and TOC1 ubiquitination in response to low temperatures, we used a proteomics approach to identify proteins involved in their ubiquitination at lower temperatures. *35Sp:TOC1-F* plants were grown under constant light at 22°C for 7 days and then subjected to MG132 treatment at 12° or 28°C for 24 hours to inhibit protein degradation. The protein extracts from the *35Sp:TOC1-F* seedlings were immunoprecipitated with the anti-FLAG antibody. Then, immunoprecipitated fractions that were enriched for TOC1-interacting proteins were analyzed by mass spectrometry (MS). PRR5-interacting proteins were isolated and analyzed using the same experimental procedure. This led to the identification of 3150 and 2804 proteins in the TOC1- and PRR5-immunoprecipitated fractions, respectively (table S1). The peptide counts from bait proteins TOC1 and PRR5 showed no statistical difference between plants incubated at 12° and 28°C due to the inhibition of 26*S* proteasome by MG132 (Fig. 3, A and B). Our results also showed that PRR5 and TOC1 interact with each other, as previously demonstrated (*25*), but the ambient temperature did not affect their interaction in vivo.

When we compared proteins enriched in the TOC1-immunoprecipitated fractions from seedlings incubated at 12° and 28°C, we identified 19 and 2 proteins that are statistically enriched in the 12° and 28°C samples, respectively [false discovery rate (FDR) < 0.01] (Fig. 3, A and C, and table S2). In the PRR5-immunoprecipitated dataset, eight and six proteins were enriched in the 12° and 28°C samples, respectively (FDR < 0.01) (Fig. 3, B and C, and table S3). LKP2 (AT2G18915) and AAA-type ATPase family protein (AT4G02480) were enriched in the 12°C samples both in the PRR5- and TOC1-immunoprecipitated datasets (Fig. 3C). The abundance of the LKP2 peptide in the TOC1-F–immunoprecipitated fraction at 12°C was eight times higher than that at 28°C (Fig. 3A). Likewise, the number of LKP2 peptides in the PRR5-F–immunoprecipitated fraction at 12°C was 3.7-times higher than that at 28°C (Fig. 3B). ACYL-ACYL CARRIER PROTEIN (ACP) DESATURASE 6 (AAD6, AT1G43800) was the only protein enriched at 28°C both in the PRR5- and TOC1-immunoprecipitated datasets (Fig. 3C).

LKP2 belongs to the ZTL/LKP2/FKF1 (FLAVIN-BINDING, KELCH REPEAT, F-BOX 1) family, a component of the E3 ubiquitin ligase SCF complex. LKP2 was shown to interact with PRR5 and TOC1 in a yeast two-hybrid assay, and the LKP2 decoy (LKP2 lacking the F-box domain to prevent ubiquitination of the targets) interacts with PRR5 and TOC1 in vivo (*26*, *27*). Accumulation of ZTL in immunoprecipitated fractions was comparable at low and high temperatures, and FKF1 was not detected in any samples (Fig. 3, A and B, and data S1 to S3).

**Fig. 4.**
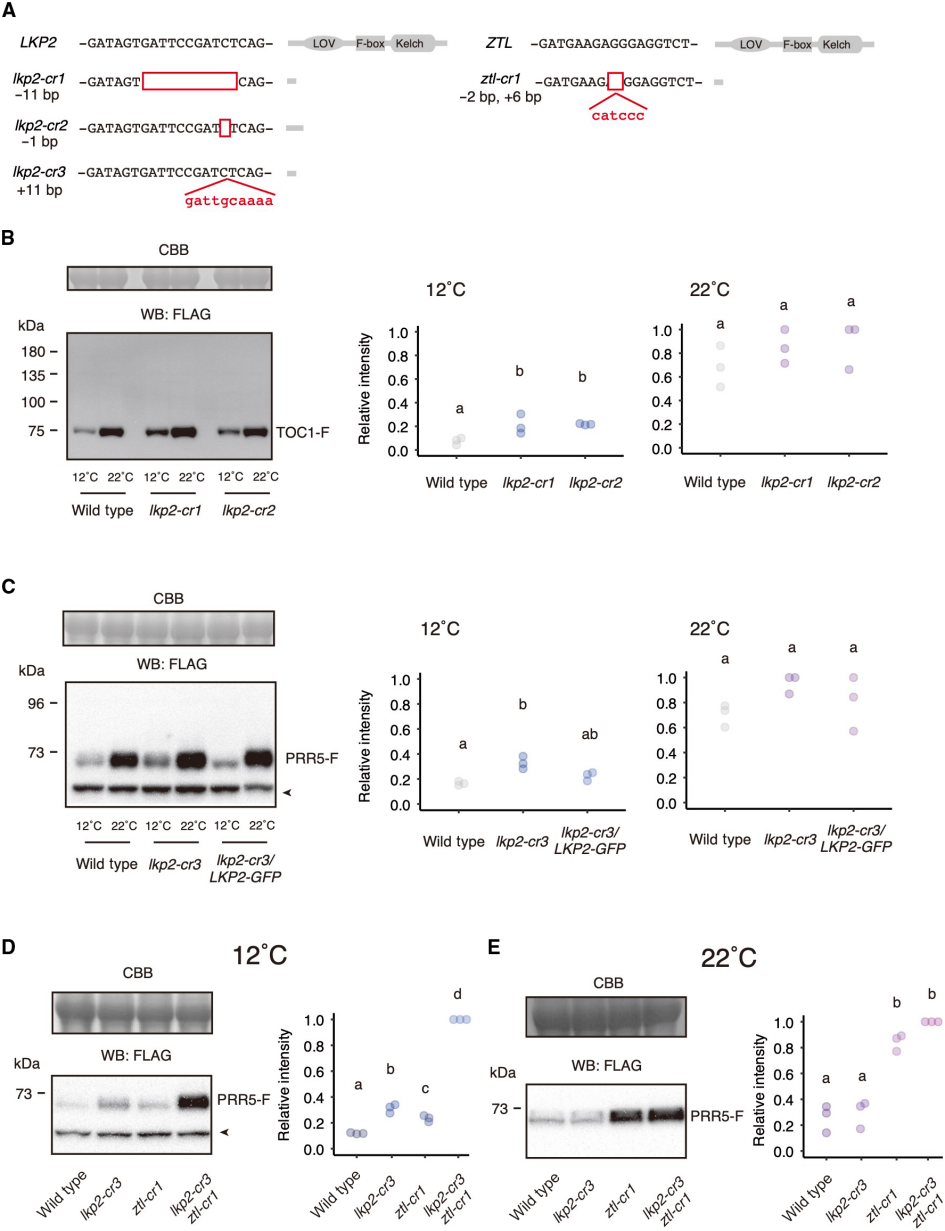
Effects of low temperature on PRR5 and TOC1 in the *lkp2* mutants. (**A**) *lkp2* mutations were created in *35Sp:TOC1-F* (*lkp2-cr1* and *lkp2-cr2*) or *35Sp:PRR5-F* plants (*lkp2-cr3*). A *ztl* mutation was created in *35Sp:PRR5-F* (*ztl-cr1*). Empty boxes indicate deletions. Prediction of LKP2 and ZTL proteins in the mutants. (**B**) TOC1-F protein accumulation in wild type and *lkp2* mutants at 12° and 22°C (left). Three independent biological replicates were used for the quantification (right). (**C**) PRR5-F protein in wild type, *lkp2-cr3*, and *lkp2-cr3/LKP2-GFP* complementation lines (right), and quantification data from three independent replicates (right). (**D**) PRR5-F protein in wild type, *lkp2-cr3*, *ztl-cr1*, and *lkp2-cr3/ztl-cr1* at 12°C. (**E**) PRR5-F protein in wild type, *lkp2-cr3*, *ztl-cr1*, and *lkp2-cr3/ztl-cr1* at 22°C. Arrowheads show nonspecific bands. The band intensity exhibiting the strongest signal in the gel was normalized to 1. Letters in the quantification graphs indicate statistical differences as determined by Tukey-Kramer test. bp, base pair.

It is possible that up-regulation of *LKP2* expression in the tested plant materials might have caused an increase of LKP2 peptides in the immunoprecipitated samples at 12°C compared to 28°C. To test this hypothesis, we examined *LKP2* mRNA in *35Sp:TOC1-F* and *35Sp:PRR5-F* plants and found that *LKP2* expression was not significantly altered by temperature (fig. S5). Next, we analyzed LKP2-GFP protein amounts in the *35Sp:PRR5-F* line (*35Sp:PRR5-F*/*lkp2-cr3*/*LKP2-GFP*; see below, Fig. 4) expressing a functional LKP2-GFP protein. We observed that the amounts of *LKP2* mRNA in *PRR5-F*/*lkp2-cr3*/*LKP2-GFP* were approximately 8 to 10 times higher than in *PRR5-F* and *PRR5-F*/*lkp2-cr3*. This suggests that *LKP2-GFP* was expressed at higher levels than the native *LKP2* (fig. S5). The amount of LKP2-GFP in *35Sp:PRR5-F*/*lkp2-cr3*/*LKP2-GFP* was 2.0-fold higher at 28°C compared to 12°C, suggesting that the difference in the total amount of LKP2 protein was unlikely the reason for the enhanced interaction between PRR5 and LKP2 at low temperature (fig. S5). We further examined the temperature-dependent interaction between LKP2-GFP and PRR5-F in vivo (Fig. 3D). The *35Sp:PRR5-F*/*lkp2-cr3*/*LKP2-GFP* seedlings were initially grown at 22°C under constant light for 7 days and further incubated at either 12° or 28°C for 24 hours with MG132 treatment to inhibit degradation of PRR5-F at 12°C. The LKP2-GFP protein was immunoprecipitated using anti-GFP antibodies. While the LKP2-GFP protein amounts were comparable in the immunoprecipitated fractions from seedling at 12° and 28°C, co-immunoprecipitated PRR5-FLAG protein from seedlings incubated at 12°C were increased 15.9-fold compared to that in seedlings at 28°C (Fig. 3D). These data support the conclusion that LKP2 interacts with PRR5 preferentially at the lower temperatures in vivo.

LKP2 is known to bind to PRR5 and TOC1, but its effect on PRR5 and TOC1 protein levels in vivo has been considered to be minor compared to that of *ZTL* (*28*). To determine whether *LKP2* regulates the low temperature–dependent decrease of PRR5 and TOC1, *lkp2* CRISPR-CAS9 mutants were generated in the background of *35Sp:TOC1-F* and *35Sp:PRR5-F* plants (Fig. 4A). TOC1-F protein amounts in *lkp2-cr1* and *lkp2-cr2* seedlings grown at 22°C were similar to those in the parental *35Sp:TOC1-F* line (*LKP2* wild type) (Fig. 4B). However, TOC1-F amounts in the *lkp2* mutants were approximately threefold higher compared to the parental line at 12°C (Fig. 4B). PRR5-F amounts in *lkp2-cr3* were comparable to the parental *35Sp:PRR5-F* line at 22°C, whereas PRR5-F protein amounts in *lkp2-cr3* were about 1.4-fold higher than in the parental line at 12°C (Fig. 4C). The up-regulation of PRR5-F in *lkp2-cr3* at 12°C was suppressed by the own promoter-driven genomic *LKP2* fused with *GFP* (*LKP2-GFP*), although PRR5-F amount in *lkp2-cr3/LKP2-GFP* was slightly higher than that in wild type (Fig. 4C). *PRR5* mRNA levels were comparable in *35Sp:PRR5-F*, *35Sp:PRR5-F*/*lkp2-cr3*, and *35Sp:PRR5-F*/*lkp2-cr3*/*LKP2-GFP* (fig. S6). Collectively, these results suggest that *LKP2* controls TOC1 and PRR5 protein amounts at lower temperatures.

Given that ZTL interacts with PRR5 both at 12° and 28°C (Fig. 3) and is known as the major factor in the degradation of PRR5 at temperatures over 22°C (*28*), we asked whether *ZTL* is involved in low temperature–dependent control of PRR5 amounts. We generated *ztl-cr1*, a CRISPR-CAS9 mutant in the background of *35Sp:PRR5-F* plants (a possible loss-of-functional mutant due to a premature stop codon in the LOV domain, Fig. 4A), and tested PRR5 protein amounts in the mutant (Fig. 4, D and E). At 22°C, PRR5-F protein amounts were increased 3.3 times in *ztl-cr1* compared to the parental line (Fig. 4E), consistent with a previous study that demonstrated ZTL-dependent PRR5 degradation (*28*). PRR5-F amounts in *ztl* and *lkp2 ztl* were comparable at 22°C, indicating that the contribution of *LKP2* to the control of PRR5-F protein amounts at 22°C is minimal even in plants lacking *ZTL*. When PRR5-F amounts were examined at 12°C, both *lkp2* and *ztl* mutations caused an increase in PRR5-F compared to the parental line (2.6- and 2.0-fold up, respectively), suggesting that *LKP2* and *ZTL* parallelly controls PRR5 protein amount at 12°C (Fig. 4D). The PRR5-F protein highly accumulated (8.4-fold up) in the *lkp2 ztl* mutant, suggesting an additive or synergistic genetic interaction between *LKP2* and *ZTL* for PRR5 protein decrease at 12°C.

### *LKP2* maintains period length at lower temperatures

**Fig. 5.**
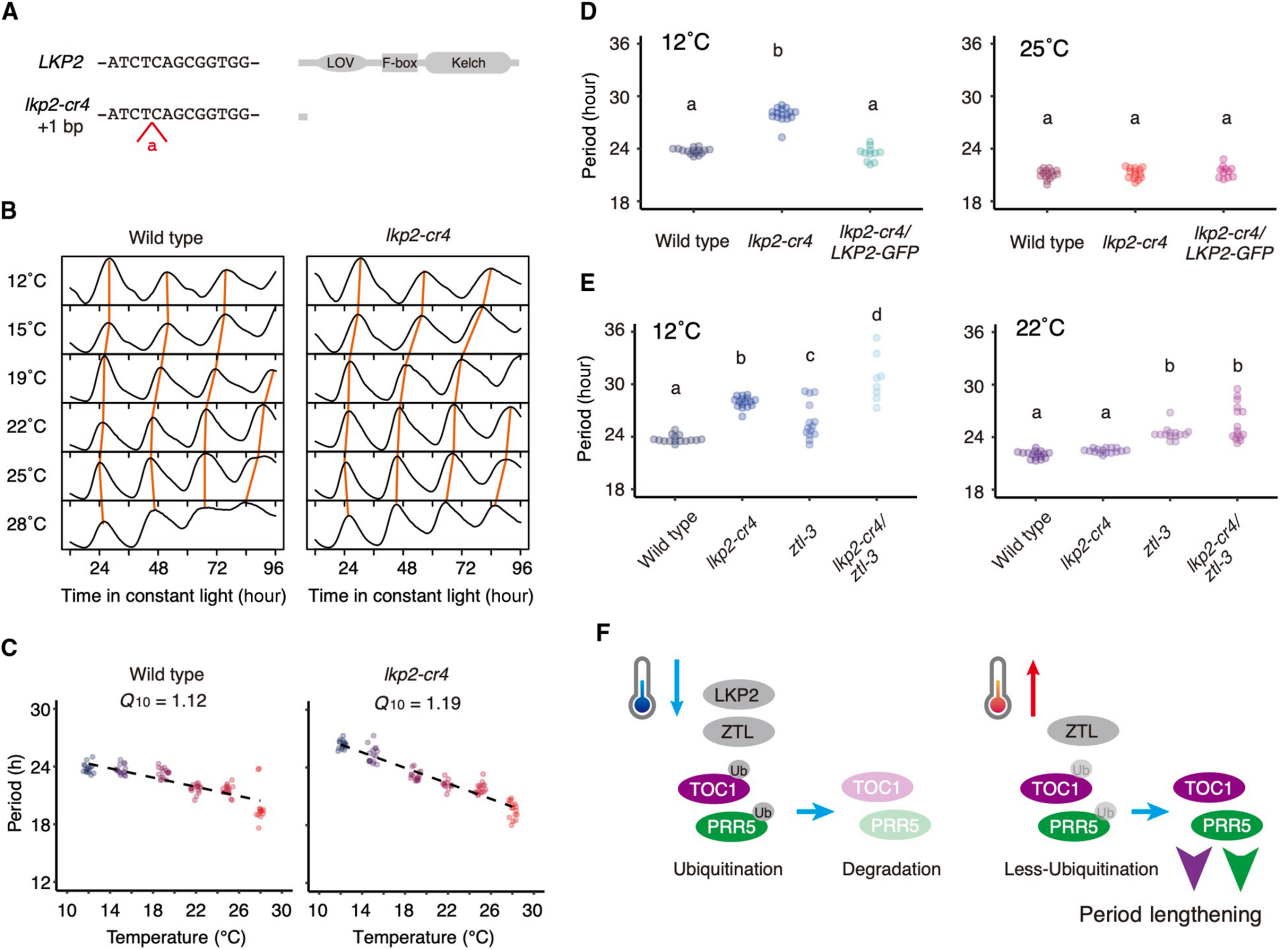
Temperature dependency of circadian period in *lkp2* mutants. (**A**) *lkp2-cr4* mutant were created in *CCA1:LUC* (left). Prediction of LKP2 protein in *lkp2-cr4* (right). (**B**) Mean traces of *CCA1:LUC* reporter (*n* = 16). (**C**) Period length and *Q*_10_ value (*n* = 13 to 16). (**D**) Comparisons of period lengths among wild type, *lkp2-cr4*, and *lkp2-cr4/LKP2-GFP* (*n* = 15 for wild type, *n* = 16 or 14 for *lkp2-cr4*, and *n* = 11 for *lkp2-cr4/LKP2-GFP*). (**E**) Comparisons of period lengths among wild type, *lkp2-cr4*, *ztl-3*, and *lkp2-cr4/ ztl-3* (*n* = 15 or 16 for wild type, *n* = 16 for *lkp2-cr4*, *n* = 14 for *ztl-3*, and *n* = 8 or 14 for *lkp2-cr4/ztl-3*)*.* Significant differences were determined using the Tukey-Kramer test. (**F**) Schematic model of the molecular basis of temperature compensation by PRR5 and TOC1. PRR5 and TOC1 are targeted for degradation by LKP2 and ZTL at lower temperatures and by ZTL at high temperatures.

The circadian rhythm of the *lkp2* single mutant was comparable to the wild type at 22°C (*28*). The *lkp2* mutation did not affect the period even in the *ztl* mutant at 22°C. Only a reduced amplitude was detected in *ztl fkf1 lkp2* compared to *ztl fkf1*, suggesting a lesser role for *LKP2* in the clock function compared to *ZTL* at 22°C (*28*). Increased PRR5 and TOC1 amounts in the *lkp2* mutant compared to the wild type at 12°C (Fig. 4) led us to examine the contribution of *LKP2* to the circadian clock at various temperatures (Fig. 5). CRISPR-CAS9–mediated genome editing in *CCA1:LUC* generated *lkp2-cr4*. *lkp2-cr4* has a nonsense codon in the region of the LOV domain (Fig. 5A). The *lkp2-cr4* mutant has comparable circadian periods to wild type at higher temperature, but at 15°C, their periods are 1.7 hours longer than the wild type (Fig. 5, B and C, and fig. S7). The periods of the *lkp2-cr4* mutant were 2.6 hours longer than wild type’s at 12°C. The *Q*_10_ value of *lkp2*-*cr4* was 1.19, significantly higher than wild type (Fig. 5C). Long period at 12°C in *lkp2-cr4* was complemented by its own promoter-driven genomic *LKP2* (*lkp2-cr4/LKP2-GFP*) (Fig. 5D). Period length in *lkp2-cr4/LKP2-GFP* was comparable to that in the wild type and *lkp2-cr4* at 25°C.

We next evaluated the genetic interaction between *LKP2* and *ZTL* with respect to period length at various temperatures. The *ztl* mutants showed a long period in all temperature conditions tested (1.7 to 3.6 hours longer than the wild type; Fig. 5E and fig. S1). *ztl* and *lkp2 ztl* showed similar circadian periods at 19°, 22°, and 28°C (Fig. 5E and fig. S1), suggesting that *ZTL* has a more critical role in the period regulation than *LKP2* within this temperature range. However, at 15° and 12°C, *lkp2 ztl* exhibited a significantly longer period compared to the single mutants *lkp2* and *ztl*. Average period lengths of *lkp2*, *ztl*, and *lkp2 ztl*, at 15°C were 25.7, 27.1, and 27.8 hours, respectively. At 12°C, the average period lengths of *lkp2*, *ztl*, and *lkp2 ztl* were 27.9, 26.0, and 30.6 hours, respectively. These results suggest that *LKP2* and *ZTL* have period-shortening roles at lower temperatures.

## DISCUSSION

In this study, we found that the regulation of PRR5 and TOC1 abundance is an essential component of temperature compensation in the *Arabidopsis* circadian clock. PRR5 and TOC1 quantities are regulated in response to variations in ambient temperature through ubiquitination and degradation (Fig. 5F). The characteristics of PRR5 and TOC1 meet the criteria for “temperature-activated period-lengthening factors” hypothesized by Hastings and Sweeny about seven decades ago, in which the activity of period-lengthening reactions balance the temperature-dependent reactions in the circadian clock (*4*). *CCA1* and its closest homolog *LATE ELONGATED HYPOCOTYL* (*LHY*) also act as period-lengthening factors at 22°C (*14*, *29*). In our study, the period length in *cca1 lhy* were determined at only 19°, 22°, and 25°C because the rhythm was severely damped at the other temperatures (fig. S1). The *Q*_10_ value of *cca1 lhy* estimated by the limited temperature range was 1.13. Given that CK2 phosphorylation of CCA1 and LHY affects temperature compensation (*13*), and amounts of CCA1 and LHY proteins were reduced at 4°C (*30*), we do not exclude the possibility that CCA1 and LHY meet for ‘temperature-activated period-lengthening factors’ for temperature compensation. In addition to the “temperature-activated period-lengthening factors PRR5 and TOC1,” the extended periodicity observed in *rve8 rve6 rve4* at lower temperatures implies that these RVE transcriptional activators function as “temperature-inactivated period-shortening factors” (*14*). This indicates that a proper balance of these temperature-responsive core clock proteins is crucial for temperature compensation of period length.

We found that *LKP2* controls the period length preferentially at lower temperatures (12° and 15°C; Fig. 5). LKP2 was thought to be a very minor clock component because *lkp2* loss-of-function mutants show only a subtle phenotype at 22°C (Fig. 5) (*28*). *LKP2* affects PRR5 and TOC1 protein amounts at 12°C, but not at 22°C, suggesting that *LKP2* is involved in the degradation of PRR5 and TOC1 only at lower temperatures (Fig. 4). We propose that low temperature–dependent PRR5-LKP2 and TOC1-LKP2 interactions (Fig. 3) are the basis for the *lkp2* phenotypes expressed specifically at low temperatures. How the temperature-dependent interactions are established is an intriguing question that remains open. High temperatures (28°C) up-regulate PRR5 and TOC1 amounts compared to 22°C, suggesting that these proteins are controlled over a broad range of temperatures. We hypothesized that the quantity control of PRR5 and TOC1 at higher temperatures is mediated by LKP2-independent pathways.

LKP2 is phylogenetically close to ZTL. *ZTL* is required for period maintenance across all temperatures tested (12° to 28°C), resulting in similar *Q*_10_ values between the *ztl* mutant and wild type (fig. S1). PRR5 and TOC1 interact with ZTL regardless of temperature (Fig. 3, A and B). *ZTL* is required for PRR5 reduction at both 12° and 22°C (Fig. 4, D and E). These data suggested that *ZTL* essentially functions at the temperature range of 12° to 28°C. What is the molecular basis for the difference in temperature response between LKP2 and its closely related protein ZTL? To explore this from an evolutionary perspective, we performed the phylogenetic analysis of ZTL/LKP2/FKF1 proteins from dicots, monocots, and *Marchantia polymorpha* using the automatic web tool ORTHOSCOPE (fig. S8) (*31*). The analysis revealed that the FKF1 clade and the ZTL/LKP2 clade diverged before the divergence of monocots and dicots. Within the ZTL/LKP2 clade, there are three subclades: monocot ZTL/LKP2 subclade, dicot ZTL subclade, and dicot LKP2 subclade. The dicot LKP2 subclade includes proteins exclusively from Brassicaceae, suggesting that this subclade diverged after the emergence of Brassicaceae (fig. S8).

To investigate the temperature dependency of LKP2 from a structural perspective, we compared the structures of ZTL and LKP2 predicted by AlphaFold2 (fig. S9) (*32*). Superimposition of full-length ZTL and LKP2 revealed structural differences of these proteins, likely due to disordered regions. Although the LOV domain, F-box, and Kelch repeats of ZTL and LKP2 showed high structural similarities, these predictions suggest that structural differences between ZTL and LKP2 may contribute to the functional differences in temperature responses. Further in-depth molecular and biochemical experiments are needed to elucidate the exact molecular basis for LKP2’s temperature response. Notably, compared to ZTL, the LKP2 LOV domain has a Q154L substitution that may attenuate blue light signaling, as similar substitutions in other LOV proteins have been shown to abolish blue light signaling (*33*), although the LKP2 LOV domain can absorb blue light in vitro (*34*).

We noticed that the period length of *ztl* at 12°C was shorter than that at 15°C (fig. S1). The slight overcompensation phenotype of *ztl* at the lower temperature was canceled by the *lkp2* mutation (fig. S1), implying that *ZTL* interferes with the period-shortening function of *LKP2* at 12°C.

Previous studies suggest that ambient temperature regulates core clock proteins in *Arabidopsis*. Relatively high temperatures (27° or 35°C) alter the formation of EARLY FLOWERING 3 speckles in vivo (*35**,* *36*). Low temperature (4°C) caused degradation of CCA1 and LHY and induced nuclear localization of RVE4 and RVE8 (*30*). Mild low temperature (16°C) reduces GI protein amount (*37*). Our finding that PRR5 and TOC1 proteins are ubiquitinated and degraded at lower temperatures adds a new posttranslational modification step to the regulation of core clock proteins in response to temperature.

PRR5 and TOC1 protein expression peaks in the evening when temperatures drop, sometimes precipitously, in the field (*38**,* *39*). Thus, it is reasonable to assume that plants promptly regulate PRR5 and TOC1 degradation without relying on transcriptional controls in response to low temperatures. Such a rapid temperature-dependent control of PRR5 and TOC1 abundance helps plants maintain their circadian period even under rapidly fluctuating temperature conditions. Recently, it was shown that blue light modulates the activity of morning-expressed PRR9 via the blue light sensor CRY2 (*40*). Therefore, PRR proteins fine-tune the circadian period in response to two major environmental cues: light and temperature.

Previous studies suggested that LKP2 binds to GI and CYCLING DOF FACTOR (CDF), key regulatory proteins in the photoperiodic flowering pathway (*41**,* *42*). It is possible that LKP2 controls flowering time via binding to GI and CDF at lower temperatures. Investigations of potential interactors of LKP2, aside from PRR5 and TOC1, may reveal the molecular mechanisms integrating external temperature and internal timing information to regulate rhythmic biological processes, which are essential components of plant physiology (*43*).

## MATERIALS AND METHODS

### Plant materials

*Arabidopsis thaliana* (*Arabidopsis*) accession Col-0 was used as the wild type. Col-0–harboring *CCA1:LUC* reporter (*44*), *prr5 toc1 CCA1:LUC* (*45*), *prr5 CCA1:LUC* (*44*), *toc1 CCA1:LUC* (*46*), *ztl CCA1:LUC* (*45*), *prr9 prr7 CCA1:LUC* (*44*), and *cca1 lhy CCA1:LUC* (*46*) were reported previously*.* The *gi-2* mutant (*47*) was transformed with *CCA1:LUC*, giving a *gi-2 CCA1:LUC* that had a short period at 22°C, as previously demonstrated (*48*). *35Sp:PRR5-FLAG (F)* (*19*) and *35Sp:GFP* (*49*) were reported previously. To generate *35Sp:TOC1-FLAG (F)*, the *TOC1* coding sequence amplified from a cDNA pool and 3xFLAG were cloned into binary vector pSK1 (*50*). pSK-TOC1-F plasmid was transformed into Col-0 via an *Agrobacterium*-mediated method (*51*). T1 lines expressing TOC1-F protein showed a short hypocotyl phenotype that was reported previously (*52*). A representative *35Sp:TOC1-F* T3 line was used in this study. *35Sp:TOC1-YFP* plants were generated though similar procedure. To generate *35Sp:TOC1-F/lkp2*, DNAs corresponding to two guide RNAs for CRISPR-Cas9–mediated genome editing of *LKP2* were cloned into pKIR1.1 (*53**,* *54*). The resulting pKIR1.1-LKP2 was transformed into *35Sp:TOC1-F* plants via an *Agrobacterium*-mediated method. Mutations in *LKP2* in plants were confirmed by Sanger sequencing as shown in Fig. 4. *35Sp:PRR5-F*/*lkp2* and *CCA1:LUC*/*lkp2* were generated using similar methods. For the genomic complementation test of *LKP2*, a region containing the *LKP2* promoter and coding sequence was cloned into pBA-PF5-GFP binary vector (*55*), resulting in pBA-PF5-LKP2-GFP. The pBA-PF5-LKP2-GFP vector was transformed into *35Sp:PRR5-F*/*lkp2cr-3* or *CCA1:LUC/lkp2-cr4*. Two independent *35Sp:PRR5-F*/*lkp2cr-3*/*LKP2-GFP* lines complemented decreased PRR5-F protein of *35Sp:PRR5-F/lkp2cr-3* at low temperature*.* Two independent *CCA1:LUC/lkp2-cr4*/*LKP2-GFP* lines complemented long periodicity of *CCA1:LUC/lkp2-cr4* at 12°C. To generate *35Sp:PRR5-F/ ztl*, DNAs corresponding to two guide RNAs for *ZTL* were cloned into pKIR1.1 as described above. The resulting pKIR1.1-ZTL was transformed into *35Sp:PRR5-F* plants via an *Agrobacterium*-mediated method. Mutations in *ZTL* in plants were confirmed by Sanger sequencing.

### Luminescence-based circadian rhythm assays

A luminescence-based circadian rhythm assay was performed as previously described (*45*) with minor modifications. Assays were performed in the growth chamber under constant temperature conditions between 12° and 28°C. Period lengths were determined as previously demonstrated (*45*). The *Q*_10_ value was calculated as a coefficient of the linear model for log-scaled frequency and temperature. To statistically compare *Q*_10_ values, we applied analysis of covariance (ANCOVA) model using R function, “*lm()*” and “*anova()*.” Note that the *Q*_10_ of *cca1 lhy* and *prr7 prr5* were determined within 19° to 25°C and 15° to 25°C range, respectively.

### Gene expression analysis

Col-0, *35Sp:TOC1-F*, and *35Sp:PRR5-F* were grown on half-strength Murashige and Skoog (MS) medium containing 1% sucrose and 0.3% gellan gum under 12-hour light/12-hour dark conditions for 7 days. Seedlings were then transferred into constant light and kept for 24 hours at 12°, 22°, or 28°C. Seedlings were collected in a tube with zirconia beads and flash-frozen in liquid nitrogen (Fig. 2A and fig. S2). *35Sp:TOC1-F* and *35Sp:PRR5-F* plants were grown on half-strength MS medium containing 1% sucrose and 0.3% gellan gum under constant light conditions at 22°C for 4 days and then transferred to constant light at 12°, 22°, or 28°C. Seedlings were sampled in a tube 24, 30, and 36 hours after transfer (Fig. 2B and fig. S3). Eight to 10 seedlings were sampled as one biological replicate. Seedlings were crushed with a Tissue Lyser II (QIAGEN), and the RNA was isolated by Illustra RNAspin (Cytiva). Reverse transcription followed by quantitative polymerase chain reaction for *CCA1* was performed with the normalization gene *IPP2* (*ISOPENTENYL DIPHOSPHATE ISOMERASE 2*), as previously demonstrated (*45*).

### Hypocotyl length measurement

For hypocotyl length assays, *35Sp:TOC1-F* and *35Sp:PRR5-F* seedlings were grown on a half-strength MS plate containing 1% sucrose and 0.3% gellan gum under 8-hour light/16-hour dark at constant 12°, 22°, or 28°C for 7 days. The hypocotyl length was measured with ImageJ.

### Protein extraction and Western blotting

Seedlings were grown on a half-strength MS plate containing 1% sucrose and 0.3% gellan gum under constant light at 22°C for 4 days and then transferred to constant light at 12°, 22°, or 28°C for 24, 30, and 36 hours (Fig. 2B and fig. S4). For treatment with the proteasome inhibitor MG132, seedlings grown under constant light at 22°C for 4 days were transferred into a 1.5-ml tube with half-strength MS containing 1% sucrose and 100 μM MG132 [CS-0471, ChemScene, prepared from a 10 mM stock solution of MG132 in dimethyl sulfoxide (DMSO)] under a 0.05-MPa vacuum for 30 s and incubated at 12° or 22°C under constant light for 24 hours. In control experiments (without MG132), the same amount of DMSO (1% v/v) was added to the liquid medium. A total of 50 mg of seedlings was collected in a tube with zirconia beads and flash-frozen in liquid nitrogen. Seedlings were crushed with a Tissue Lyser II (QIAGEN). A total of 200 μl of urea lysis buffer [50 mM tris-HCl (pH 6.8), 1% SDS, 5% 2-mercaptoethanol, and 7.2 M urea] (*56*) was added to each sample, boiled for 5 min, and centrifuged for 5 min at 18,000*g*. The supernatant was loaded onto a Super Sep Ace 10% gel (192-14961, Fujifilm-Wako) and blotted onto Amersham Hybond-P polyvinylidene difluoride (PVDF) 0.45 (10600023, Cytiva). To detect FLAG fusion proteins, Anti-FLAG antibody (F3165, Sigma-Aldrich), goat anti-mouse immunoglobulin G conjugated with horseradish peroxidase (HRP) (NA931VS, Cytiva) was used as primary and secondary antibodies in 5% skim milk tris-buffered saline (TBST) buffer [5% skim milk, 20 mM tris-HCl (pH 7.5), 150 mM NaCl, and 0.1% Tween 20]. To detect GFP fusion proteins, anti-GFP antibody (04363-66, Nacalai Tesque Inc.) was used as primary antibody with Can Get Signal (NKB-101/NYPBR, TOYOBO) according to the supplier’s protocol. Peroxidase was detected by SuperSignal West pico PLUS Chemiluminescent Substrate (34577, Thermo Fisher Scientific) with a WSE-6100 LuminoGraph (ATTO Products, Tokyo, Japan). Signal intensities of Western blotting bands were measured with ImageJ.

### Detection of PRR5 and TOC1 ubiquitination

*35Sp:PRR5-F* and *35Sp:TOC1-F* were grown on half-strength MS containing 1% sucrose and 0.3% gellan gum under constant light at 22°C for 7 days and then incubated at 12° or 28°C for 1 day. About 100 seedlings were collected and flash-frozen in liquid nitrogen. Frozen samples were ground to a fine powder and suspended in lysis I buffer [50 mM tris-HCl (pH 7.5), 100 mM NaCl, 0.1% Triton X-100, 100 μM MG132, proteinase inhibitor (P9599, Sigma-Aldrich), PhosSTOP (4906845001, Merck)]. The sample was sonicated four times at 50% output for 15 s with an Ultrasonic Processor VCX-1 (Sonics Materials) and centrifuged at 18,000*g* 4°C 10 min. The supernatant was immunoprecipitated with anti-FLAG antibody (F3165, Sigma-Aldrich), which was bound to Dynabeads Protein G (10003D, Thermo Fisher Scientific) for 2 hours at 4°C. The immunoprecipitated fraction was loaded onto a Super Sep Ace 10% gel and blotted onto Amersham Hybond-P PVDF 0.45. Mouse anti-ubiquitin (UBQ) antibody (sc-8017, Santa Cruz Biotechnology) and goat anti-mouse antibody conjugated to HRP were used with Can Get Signal.

### LC/MS-MS analysis of PRR5 and TOC1 immunoprecipitation complexes

About 100 seedlings of *35Sp:PRR5-F* and *35Sp:TOC1-F* grown under constant light at 22°C for 7 days were treated with 100 μM MG132 under 0.05-MPa vacuum for 15 s and then incubated at either 12° or 28°C for 1 day. The harvested seedlings were ground in liquid nitrogen and suspended in lysis I buffer. Protein extraction and immunoprecipitation with anti-FLAG antibody were as described above. The immunoprecipitated beads were then washed with the wash buffer [50 mM tris-HCl (pH 7.5), 100 mM NaCl, 50 μM MG132, proteinase inhibitor (P9599, Sigma-Aldrich), and PhosSTOP (4906845001, Merck)] three times, suspended in 20 μl of digestion buffer (8 M urea and 250 mM ammonium bicarbonate), reduced with 25 mM tris(2-carboxyethyl)phosphine at 37°C for 15 min, and alkylated using 25 mM iodoacetamide at 37°C for 30 min in the dark, both with shaking at 1200 rpm. Proteins on the beads were directly digested with 0.1 μg of Lys-C (Fujifilm Wako, Japan) at 37°C for 3 hours with shaking at 1000 rpm. After dilution to a urea concentration of 2 M with 50 mM tris-HCl (pH 8.5), followed by addition of 1 mM CaCl_2_, the Lys-C digest was further digested with 0.1 μg of trypsin (Promega) with shaking at 1000 rpm at 37°C overnight. The digestion was stopped by adding 5 μl of 20% trifluoroacetic acid, and then Lys-C/trypsin double digested peptides were desalted using a GL-Tip SDB (GL Science) according to the manufacturer’s instructions.

Digested samples were analyzed by nano–liquid chromatography tandem mass spectrometry (LC-MS/MS) using Orbitrap Exploris 480 as previously described (*57*). MS/MS raw files were processed with Proteome Discoverer (Thermo Fisher Scientific) using the SEQUEST HT algorithm with a default setting against the TAIR10 *Arabidopsis* protein database.

### Quantitative analysis, statistics, and graph drawing

Statistical analysis was performed using R v.4.3.0 operated by RStudio v.3034.03.0+386 (*58*). Comparison between two groups was performed for Welch’s unpaired *t* test or Student’s *t* test for a pairwise comparison. One-way analysis of variance (ANOVA) followed by Tukey’s post hoc test were performed for comparison of multiple groups. Graphs were generated using the R packages, ggplot2, and ggbeeswarm.

### Phylogenic tree analysis

Phylogenic tree was generated by the automated web tool ORTHOSCOPE (*31*) with *Arabidopsis* ZTL, LKP2, and FKF1 as queries. *M. polymorpha* proteins were used as outgroups.

### Comparison of protein structure

Predicted structures of ZTL and LKP2 proteins were obtained from the AlphaFold2 database (https://alphafold.ebi.ac.uk) (*32*), with accession numbers Q94BT6 and Q8W420 for ZTL and LKP2, respectively. Whole protein structures were superimposed with UCSF Chimera (*59*) and VMD (*60*). The LOV domain (32 to 161 amino acids for ZTL and 35 to 158 amino acids for LKP2), F-box (195 to 241 amino acids for ZTL and 196 to 242 amino acids for LKP2), and Kelch repeat (292 to 609 amino acids for ZTL and 293 to 611 amino acids for LKP2) were superimposed using a similar method.
