# Supplementary material for: Cold-induced degradation of core clock proteins implements temperature compensation in the Arabidopsis circadian clock
Source: Sci Adv. 2024 Sep 27;10(39):eadq0187. doi: 10.1126/sciadv.adq0187 (PMC11430399; doi:10.1126/sciadv.adq0187)
Supplement: Supplementary file 1 — Figs. S1 to S9 Tables S1 and S2 Legends for data S1 to S3 [file sciadv.adq0187_sm.pdf]

Science Advances
AAAS

Supplementary Materials for

Cold-induced degradation of core clock proteins implements temperature compensation in the *Arabidopsis* circadian clock

Akari E. Maeda *et al.*

Corresponding author: Norihito Nakamichi, nnaka@agr.nagoya-u.ac.jp

Sci. Adv. **10**, eadq0187 (2024)
DOI: 10.1126/sciadv.adq0187

The PDF file includes:

Figs. S1 to S9
Tables S1 and S2
Legends for data S1 to S3

Other Supplementary Material for this manuscript includes the following:

Data S1 to S3

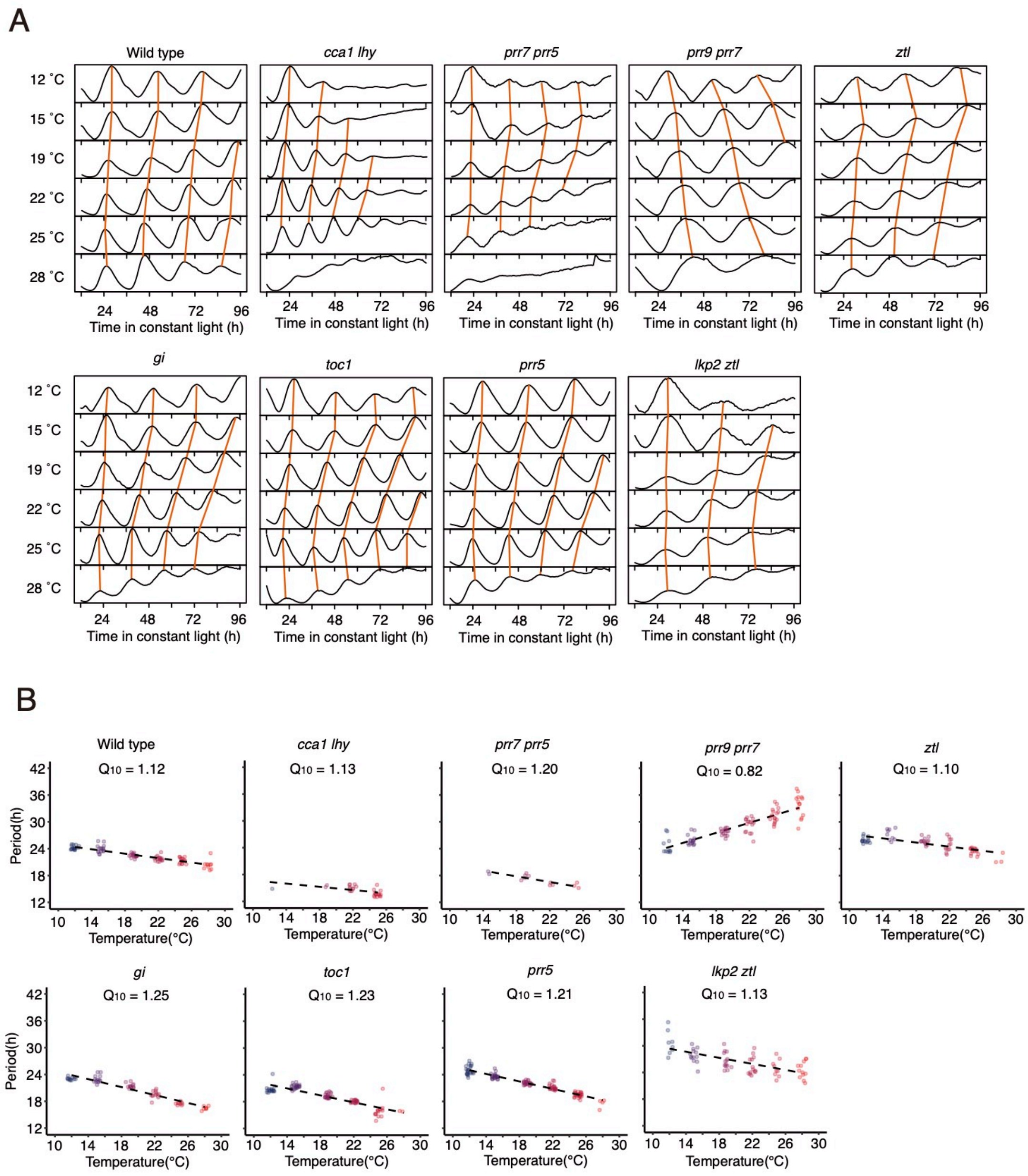

Fig. S1

Fig. S1. Circadian rhythm in period mutants at temperatures ranging from 12 to 28°C. (A), Mean traces of *CCA1:LUC* reporter in *cca1 lhy*, *prr7 prr5*, *prr9 prr7*, *ztl*, *gi*, *toc1*, *prr5, lkp2 ztl* mutants (n = 16). (B), Period length and Q_{10} value. Note that samples with fitting errors >0.05 were discarded due to aberrant period determination. Data of wild type is the same as in Figure 1.

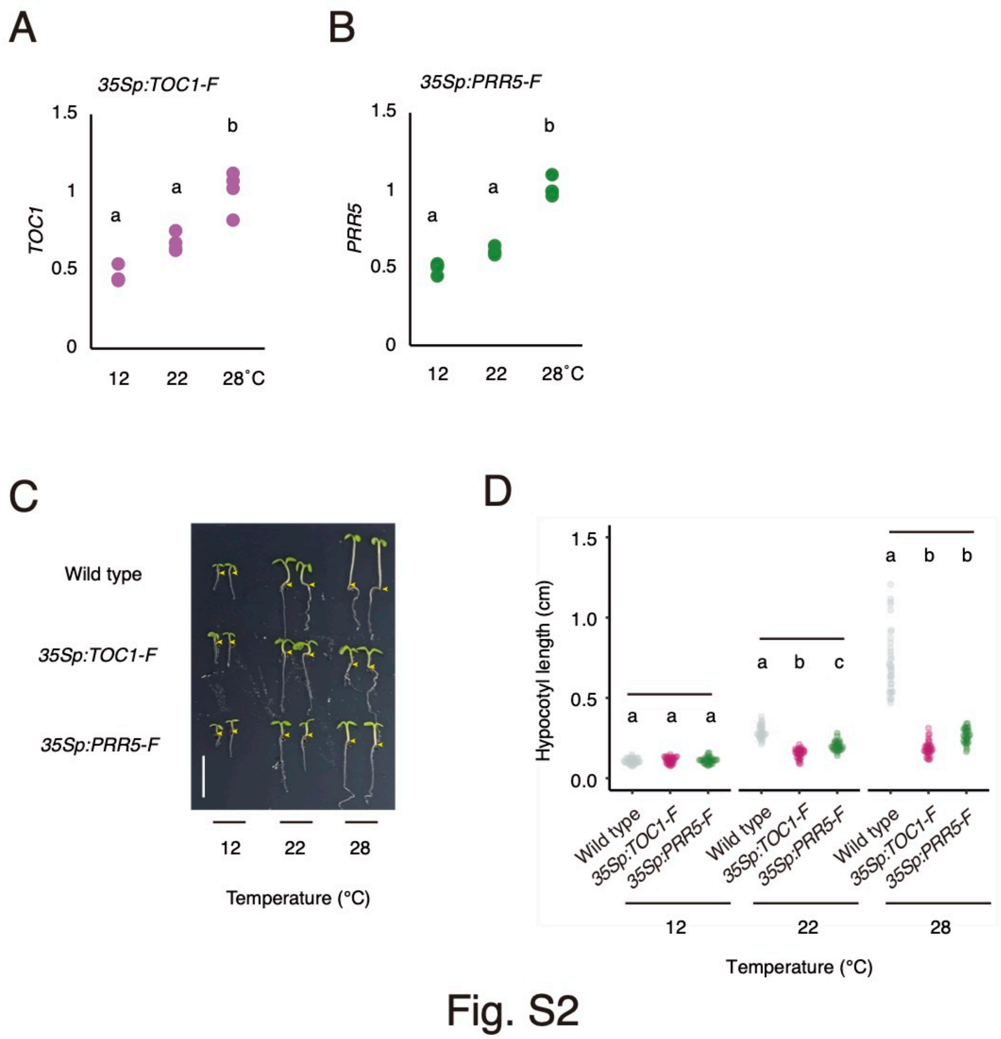

Fig. S2. *TOC1* and *PRR5* expression and hypocotyl length in overexpressors. (A), *TOC1* mRNA expression relative to *IPP2* in *35Sp:TOC1-F* at 12˚C, 22˚C, and 28˚C (four biological replicates). (B), *PRR5* mRNA expression normalized with *IPP2* in *35Sp:PRR5-F* at 12˚C, 22˚C, and 28˚C (n = 4). The mean values at 28˚C was normalized to 1.0. (C) *35Sp:TOC1-F* and *35Sp:PRR5-F* seedlings grown at 12˚C, 22˚C, or 28˚C under 8 h light/ 16 h dark conditions. White bar is 1 cm. Yellow arrowheads indicate the junction between hypocotyl and root. (D) Hypocotyl length of *35Sp:TOC1-F* and *35Sp:PRR5-F* seedlings (n = 31 ~ 38). Significant differences were determined using the Tukey-Kramer test.

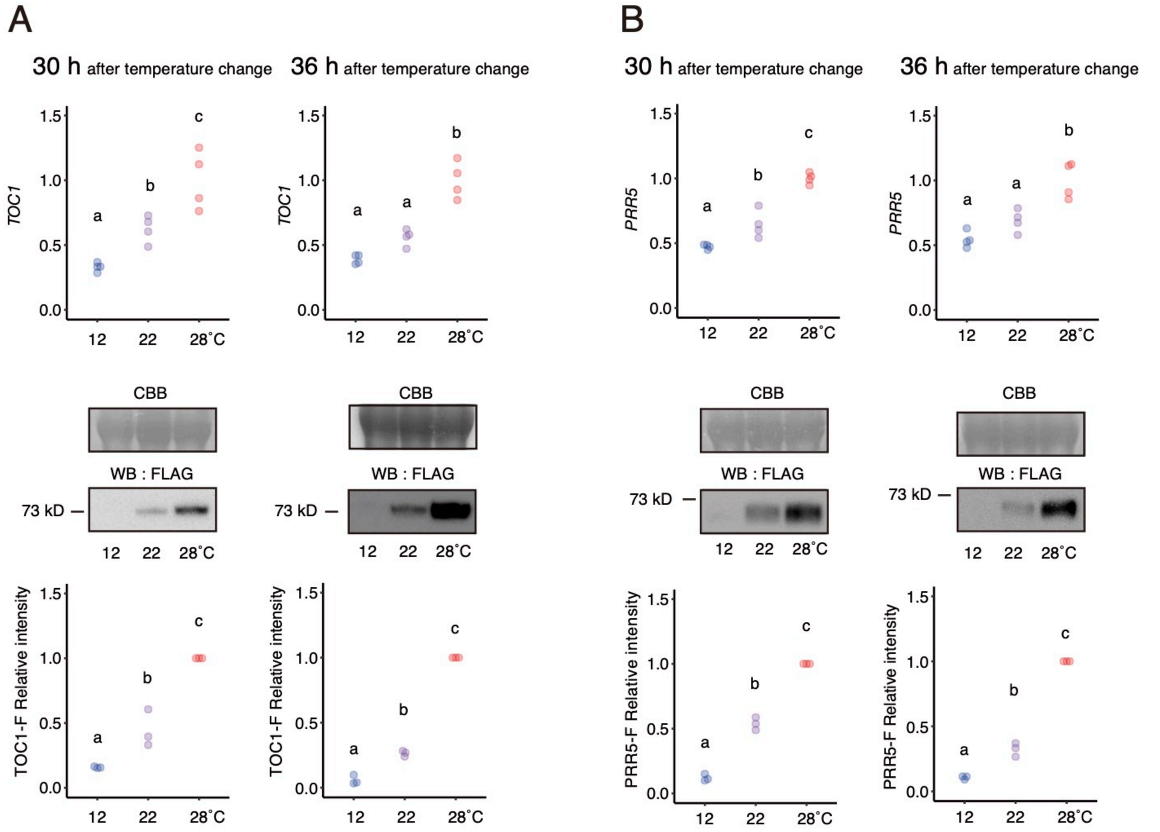

Fig. S3

Fig. S3. *TOC1*-F in *35Sp:TOC1-F* and *PRR5-F* proteins in *35Sp:PRR5-F* seedlings 30 or 36 h after temperature shifts to 12˚C, 22˚C, and 28˚C. (A) *TOC1* mRNA expression relative to *IPP2* (four biological replicates, upper) and TOC1-F protein (three biological replicates, lower) in *35Sp:TOC1-F* at 12˚C, 22˚C, and 28˚C. Typical sample of CBB staining gel and western blotting with anti-FLAG antibody are shown (middle). (B) *PRR5* mRNA (n = 4, upper) and PRR5-F protein (n = 3, lower) in *35Sp:PRR5-F* at 12˚C, 22˚C, and 28˚C. Letters in charts indicate statistical differences as determined by Tukey-Kramer test.

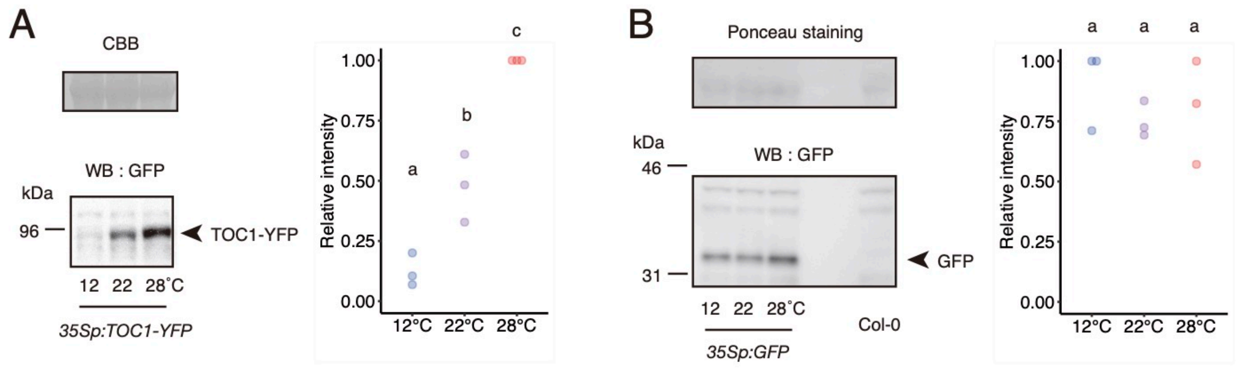

Fig. S4

Fig. S4. TOC1-YFP and GFP protein accumulation at 12˚C, 22˚C, and 28˚C. (A) TOC1-YFP protein accumulation in seedlings at 12˚C, 22˚C, and 28˚C. Left panels are Coomassie Brilliant Blue (CBB)-stained gel and western blotting with anti-GFP antibody (WB: GFP). (B) GFP protein accumulation. Left panels are ponceau staining and western blot (WB: GFP). Charts in the right were generated from three biological replicates. The band intensity with the strongest signal corresponding to TOC1-YFP (A) or GFP (B) in the gel was normalized to 1. Letters in chart indicate statistical differences as determined by Tukey-Kramer test.

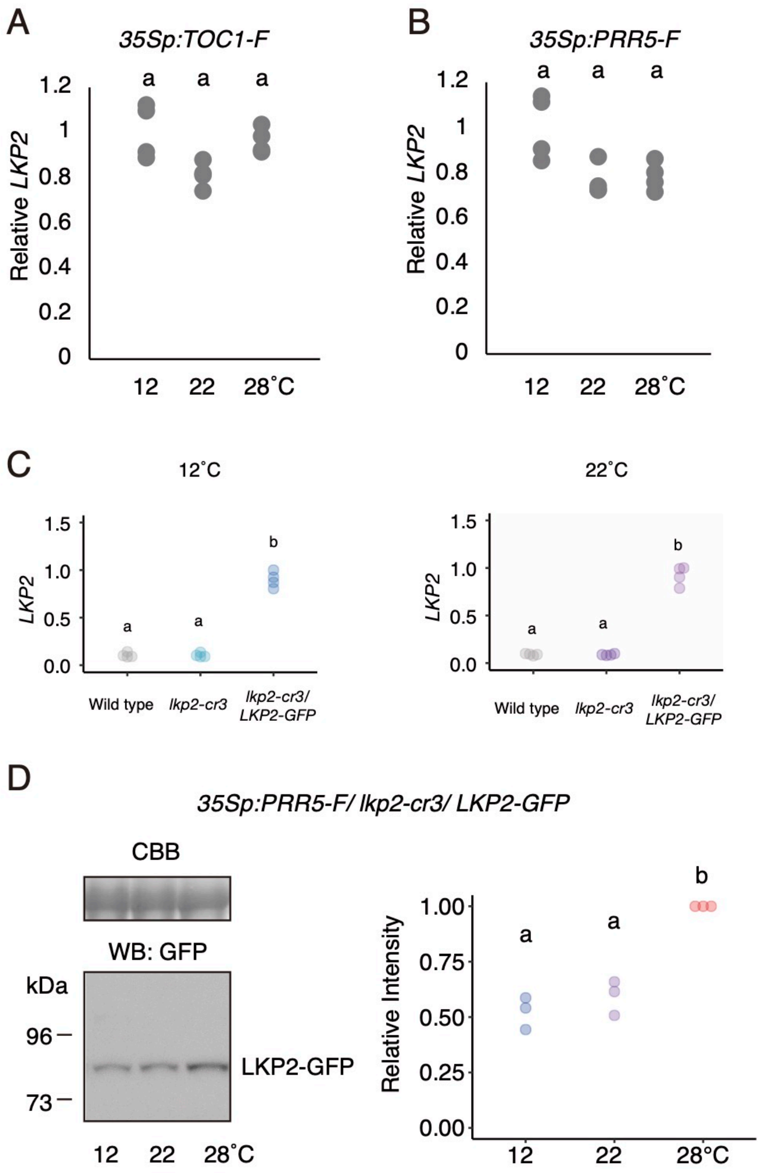

Fig. S5

Fig. S5. *LKP2* mRNA and LKP2 protein accumulation. *LKP2* mRNA normalized with *IPP2* in *35Sp:TOC1-F* (A) and *35Sp:PRR5-F* (B). Four biological replicates were used. (C) *LKP2* mRNA in *PRR5-F* (wild-type), *PRR5-F lkp2-cr3* (*lkp2-cr3*), and *PRR5-F/ lkp2-cr3/ LKP2-GFP* (*lkp2-cr3/ LKP2-GFP*) (n = 4). Seedlings grown under constant light at 22˚C were transferred at 12˚C or remained at 22˚C for 24 h. (D) LKP2-GFP protein in *35Sp:PRR5-F/ lkp2-cr3/ LKP2-GFP*. Panels on the left are Coomassie Brilliant Blue (CBB)-stained gel and western blotting with anti-GFP antibody (WB: GFP). The band intensity of LKP2-GFP at 28˚C sample was normalized to 1.0. Letters in graphs show statistical differences as determined by Tukey-Kramer test. Three biological replicates were used.

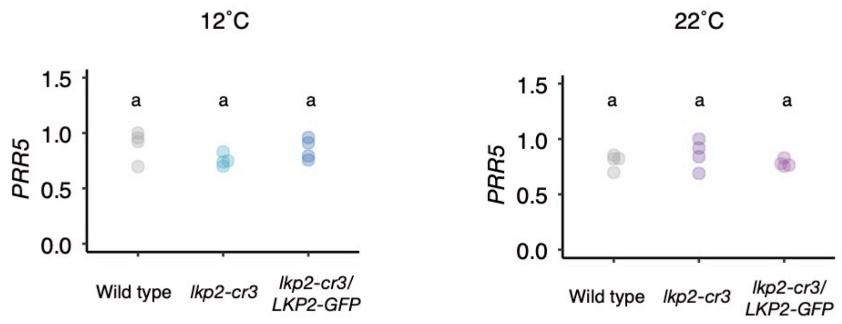

Fig. S6

Fig. S6. *PRR5* mRNA expression in *35Sp:PRR5-F* (*LKP2* wild type), *35Sp:PRR5-F lkp2-cr3*, and *35SpPRR5-F lkp2-cr3 LKP2-GFP*. Seedlings grown under constant light at 22˚C were transferred at 12˚C or 22˚C and kept there for 24 h. Letters in charts indicate statistical differences as determined by Tukey-Kramer test. Four biological replicates were examined.

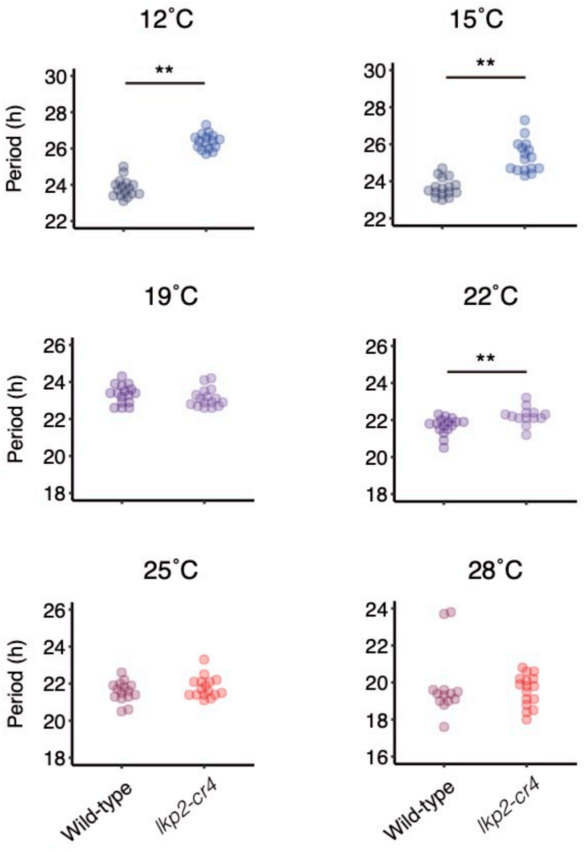

Fig. S7

Fig. S7. Comparisons of period lengths between wild type and *lkp2-cr4* (n = 13 ~ 16 for wild type, n = 13 ~ 16 for *lkp2-cr4*). Double asterisk indicates statistical differences $p < 0.01$ as determined by Student's T-test.

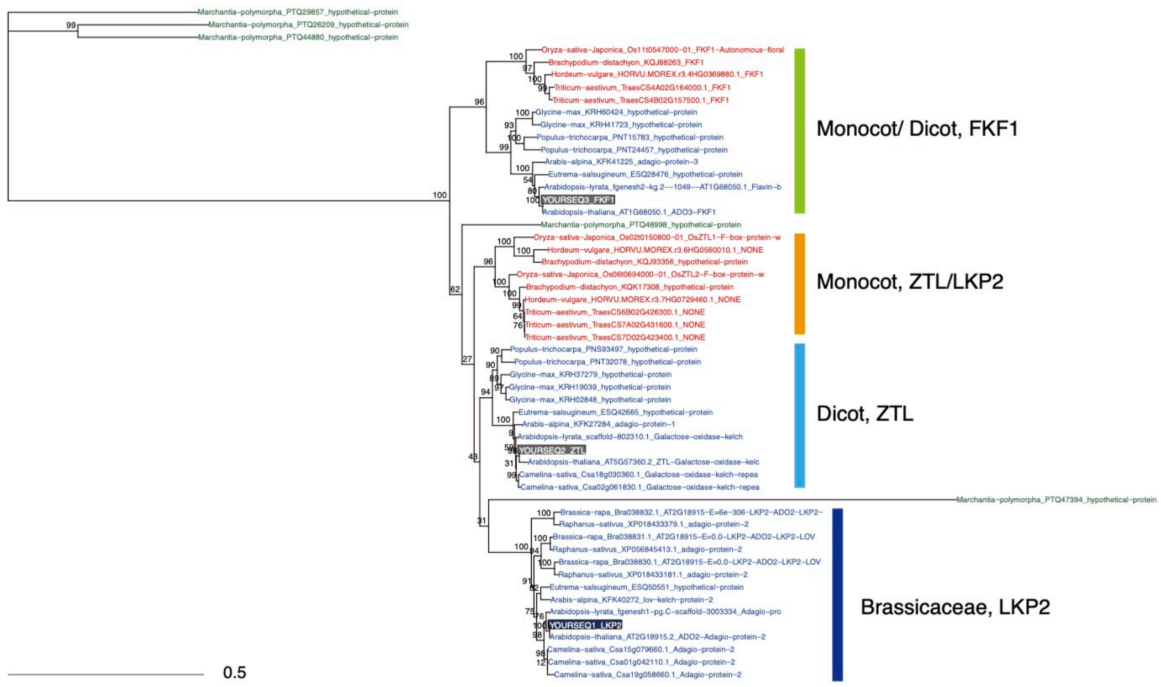

Fig. S8

Fig. S8. Phylogenetic tree of ZTL/LKF2/FKF1 in flowering plants. The phylogenetic tree was generated using automatic web tool ORTHOSCOPE with *Arabidopsis* ZTL, LKP2, and FKF1 amino acid sequences as queries. Red-colored proteins are from monocots (*Oryza sativa, Triticum aestivum, Hordeum vulgare,* and *Brachypodium distachyon*). Blue-colored proteins are from dicots (*Arabidopsis thaliana, Arabidopsis lyrata, Camelina sativa, Brassica rapa, Raphanus sativus, Eutrema salsugineum, Arabis alpina, Glycine max, Populus trichocarpa*). Green-colored proteins are from *Marchantia polymorpha*.

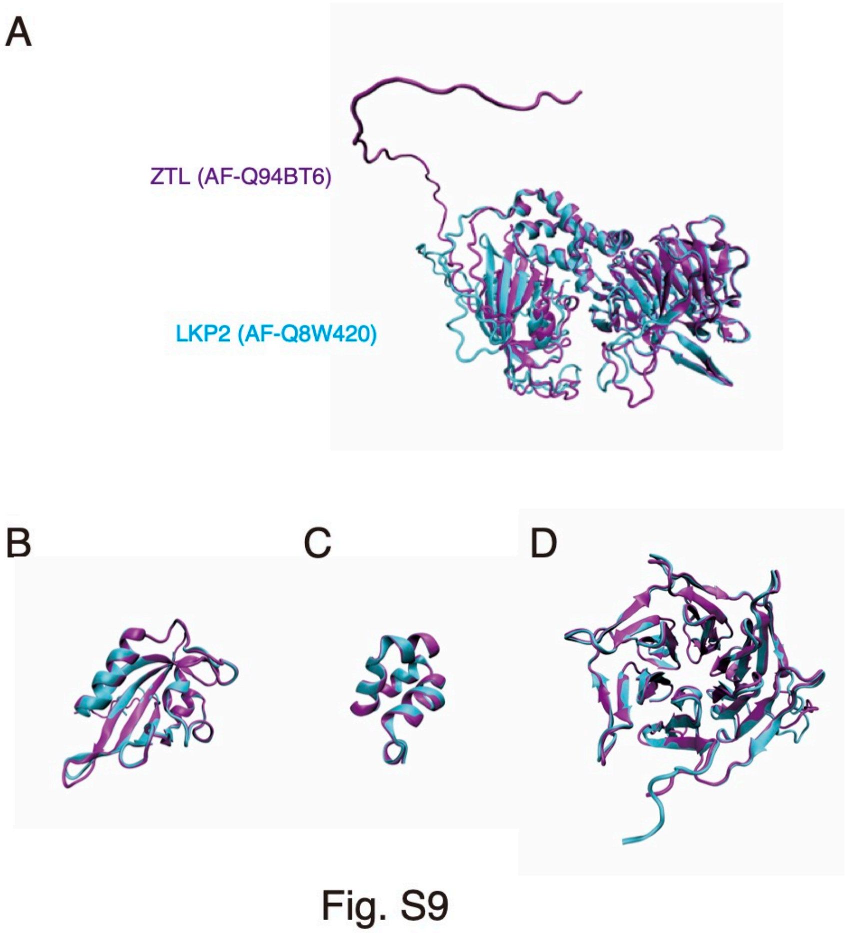

Fig. S9. Predicted structure of ZTL and LKP2. (A) ZTL (purple) and LKP (cyan) whole structures as well as their LOV domain (B), F-box (C), and Kelch repeats (D), as predicted in the AlphaFold2 database (https://alphafold.ebi.ac.uk), are shown superimposed.

genotype	period at 22˚C	Q10	Pvalue vs WT
WT (test1)	21.78	1.117	-
prr5 toc1	17.00	1.302	4.62E-25
cca1 lhy	15.02	1.134	0.548422099
prr7 prr5	16.03	1.205	0.0055306
prr9 prr7	29.29	0.823	2.58E-56
ztl	24.79	1.099	0.170686899
gi	19.53	1.249	2.86E-19
toc1	18.02	1.234	1.27E-11
prr5	21.13	1.211	2.02E-18

genotype	period at 22˚C	Q10	Pvalue vs WT
WT (test2)	21.69	1.117	-
lkp2-cr4	22.22	1.192	2.50E-09
lkp2-cr4 ztl-3	25.45	1.135	0.3156

Table S1. Period length and Q_{10} value of wild type and various mutants at 22°C.

Primers used in this study

Construction of lkp2 and ztl mutants by CRISPR/CAS9

	name	sequence (5'->3')	Restriction enzyme site
pKIR1.1-LKP2	LKP2crispr_Fw	TTGGGTCTCAATTG**CGCTGACCACAAACCCACA**GTTTTAGAGCTAGAAATAGCA	Bsa1
	LKP2crispr_Rv	TTGGGTCTCTAAAC**CTGAGATCGGAATCACTATC**TGCACCAGCCGGGAATCGAA	Bsa1
pKIR1.1-ZTL	ZTLcrispr_Fw	TTGGGTCTCAATTG**CAACCATAGAGTCAACTAA**GTTTTAGAGCTAGAAATAGCA	Bsa1
	ZTLcrispr-Rv	TTGGGTCTCTAAAC**TCTTCATCATCCGCCAGTGA**TGCACCAGCCGGGAATCGAA	Bsa1
		* DNA for guide RNA is shown in Bold.	

Construction of Plasmid for transgenic plants

	name	sequence (5'->3')	Restriction enzyme site
LKP2p:LKP2-GFP complimentation	LKP2pro_Not1_Fw	GCAGGCTCCGCGGCCGCCCCACTTGTTTGGTGGCTCTCCC	Not1
	LKP2_Asc1_Rv	AGCTGGGTCGGCGCGCCCAGTACTTGCAGTGGTAGAAGTTGCTAG	Asc1
35Sp:TOC1-YFP	TOC1_Xba1_Fw	ATCGTCTAGACATGGATTTGAACGGTGAGTGTAAAG	Xba1
	TOC1_Nco1_Rv	GCATCCATGGTAGTTCCCAAAGCATCATCCTGAG	Nco1

qPCR primer

	name	sequence (5'->3')
LKP2(AT2G18915)	LKP2_Fw_qPCR	GGTCAGCGATGCTCTTGAACCTG
	LKP2_Rv_qPCR	GGCCCTCTACATTGCAAGAAACG

Table S2. Primers used in this study

Data S1. (separate file). Peptide numbers in TOC1-F- or PRR5-F-immunoprecipitated fractions. Blue and red colored columns are peptides from 12˚C and 28˚C sample, respectively.

Data S2. (separate file). Fold and FDR changes between peptide numbers in 12˚C and 28˚C grown plants TOC1-F-immunoprecipitated fractions.

Data S3. (separate file). Fold and FDR changes between peptide numbers in 12˚C and 28˚C grown plants PRR5-F-immunoprecipitated fractions.
